# Dissociating the Disruptive Effects of Irrelevant Music and Speech on Serial Recall of Tonal and Verbal Sequences

**DOI:** 10.3389/fpsyg.2020.00346

**Published:** 2020-03-05

**Authors:** Florian Kattner, Hanna Meinhardt

**Affiliations:** ^1^Institute of Psychology, Technische Universität Darmstadt, Darmstadt, Germany; ^2^Institute of Psychology, University of Hamburg, Hamburg, Germany

**Keywords:** short-term memory, musical memory, auditory distraction, irrelevant speech effect, irrelevant music, serial recall

## Abstract

Task-irrelevant speech or music sounds are known to disrupt verbal short-term memory even when participants are instructed to ignore the sound, suggesting that automatically processed acoustical changes interfere with the rehearsal of phonological items. However, much less is known about auditory distraction in tasks that require the memorization and recall of non-phonological auditory items. In the present study, both musically trained and untrained participants were asked to memorize random tone sequences (consisting of low, medium, and high pitch tones) while task-irrelevant sound was presented. Irrelevant instrumental music was found to produce more disruption of tonal recall than white noise, whereas irrelevant speech produced intermediate levels of disruption. In contrast, only speech produced significant interference in an analogous verbal recall task. Crucially, although musically trained participants were able to recall more tones in general, the degree of auditory distraction that was produced by irrelevant music in the tonal recall task was found to be independent of musical expertise. The findings are in line with the assumption of two separate mechanisms for the maintenance of tonal and phonological information. Specifically, short-term memory for tone sequences may rely on a pitch-based rehearsal system which is disrupted by the perception of irrelevant pitch changes as contained in instrumental music (and to a lesser extent in speech), whereas serial recall of verbal items is most sensitive to phonological sounds.

## Introduction

According to the most prominent working memory model (Baddeley and Hitch, 1974; Baddeley, 1992, 2003), temporary storage of verbal information is based on subvocal articulatory rehearsal in a phonological loop which is supposed to operate independently from the mechanisms used for storage of visual and spatial information. In line with this account, it has been found that short-term memory is superior for phonologically distinct verbal items than for phonologically similar items even when the items are presented visually (Conrad, 1964; Salamé and Baddeley, 1986), and that the maintenance of verbal information is disrupted by articulatory movements (“articulatory suppression”; Baddeley et al., 1975) and task-irrelevant speech (“irrelevant speech effect”; Colle and Welsh, 1976; Salamé and Baddeley, 1982). These observations suggest that verbal information is maintained in short-term memory through phonological rehearsal (i.e., using a phonological code), with irrelevant articulatory movements and speech sounds gaining obligatory access to the phonological store. More specifically, the phonological similarity effect suggests that the poorer recall of items with similar phonological codes is due to the absence of discriminatory features, whereas the irrelevant speech effect indicates direct access of speech sounds to the phonological store, leading to interference with the to-be-remembered items (Baddeley, 1992). The working memory model further predicts that articulatory suppression attenuates the irrelevant speech effect if the to-be-remembered items are presented visually (e.g., Salamé and Baddeley, 1982; Miles et al., 1991; Divin et al., 2001; indicating that the articulatory loop is needed for grapheme-to-phoneme conversion), whereas articulatory suppression should not affect the irrelevant speech effect when both irrelevant and relevant items are auditory (see Hanley and Broadbent, 1987). Articulatory suppression, however, was found to attenuate the effect with both visual and auditory items if the perceptual segregation of relevant and irrelevant auditory streams was facilitated (see Jones et al., 2004). Likewise, if irrelevant speech gained direct access to the phonological store when the articulatory loop is occupied, then the phonological similarity effect on serial recall of auditory items (in contrast to visual items) should also persist under conditions of articulatory suppression (e.g., Murray, 1968; Baddeley et al., 1984). However, in this case, the presence of a suffix (a redundant item after presentation of the to-be-remembered list) was found to be sufficient to eliminate the phonological similarity effect with auditory items under articulatory suppression, indicating that the effect may have been driven by perceptual processes rather than by phonological rehearsal (Jones et al., 2004).

In contrast to the disruptions produced by irrelevant speech, and also in line with the assumption of a phonological store, non-speech sounds such as instrumental music were found to interfere to a lesser extent with verbal short-term memory (Salamé and Baddeley, 1989). However, there have been multiple studies in the last three decades demonstrating that serial recall of verbal items can be disrupted considerably by different types of non-speech sounds such as sequences of tones (Jones and Macken, 1993; Bell et al., 2019), pitch glides (Jones et al., 1993), sine-wave speech (Tremblay et al., 2000; Viswanathan et al., 2014), or music (Salamé and Baddeley, 1989; Schlittmeier et al., 2008; Schlittmeier and Hellbrück, 2009). Based on these and similar findings, it has been suggested that the interference between irrelevant sound and verbal serial recall is based on the presence of “changing-state” sounds (e.g., varying tones being more disruptive than repeated tones; Jones and Macken, 1993), and it has been found that the degree of auditory distraction produced by speech increases with the extent of spectral modulations (e.g., Schlittmeier et al., 2012; Ellermeier et al., 2015; Dorsi et al., 2018; Kattner and Ellermeier, 2018; Schlittenlacher et al., 2019). The “changing-state” account can thus explain the frequently reported finding that verbal short-term memory is disrupted by the presence of both irrelevant speech and irrelevant music given that it contains a certain degree of spectral fluctuations (Salamé and Baddeley, 1989; Nittono, 1997; Ellermeier and Hellbrück, 1998; Schlittmeier et al., 2008; Schlittmeier and Hellbrück, 2009; Perham and Vizard, 2011; Avila et al., 2012).

While the disrupting effect of task-irrelevant sound has been well studied extensively for verbal short-term memory, it is less clear whether short-term memory for non-phonological auditory information such as sequences of tones or music is processed by the same loop (i.e., using phonological rehearsal) or whether a different component of auditory working memory exists for the maintenance of tonal information (e.g., a “musical” or “tonal” loop; see Berz, 1995; Pechmann and Mohr, 1992; Schulze and Koelsch, 2012). If tonal information was processed by a separate auditory loop, then task-irrelevant phonological information should interfere more with verbal than with tonal short-term memory, whereas irrelevant tonal information should produce stronger disruption of tonal than of verbal short-term memory. However, in contrast to the numerous studies on verbal short-term memory (typically using a serial recall task), there are only a few studies on short-term memory of non-phonological tonal stimuli. In one early study, pitch recognition was found to be disrupted by the presence of intervening and irrelevant tones during the retention interval, whereas articulatory rehearsal of numbers and the presence of irrelevant speech were found to be much less disrupting in a pitch-recognition task (Deutsch, 1970). Several other studies, however, reported disrupting effects of both irrelevant music and speech on the recognition of tonal pitch using a similar paradigm (Pechmann and Mohr, 1992; Semal et al., 1996; Ueda, 2004), and it has been argued that the degree of distraction in a tonal recognition task depends on the proximity in pitch between the irrelevant and the to-be-remembered auditory items. Hence, it is still possible that the same or overlapping working memory mechanisms are used for encoding and maintenance of verbal and tonal stimuli. Very few studies, however, directly compared serial recall of verbal and tonal stimuli.

In one study, a tonal analog of the phonological similarity effect has been studied for serial recall of tonal sequences (Williamson et al., 2010a). Specifically, the participants of that study were asked to recall sequences of either letters or tones. The letters were either phonologically similar (B, V, G) or distinct (F, K, R) while tones were either pitch-proximal (i.e., separated by two semitones: C4, D4, E4) or pitch-distal (C4, G4, B4). The authors developed a tonal serial recall task which could be done by participants with and without musical experience: the to-be-remembered tonal sequences were composed of three to seven tones drawn with replacement from a set of only three different pitches that were labeled as “low,” “medium,” and “high.” In analogy to the phonological similarity effect on verbal serial recall, the authors found that serial recall of tones was subject to a pitch-proximity effect with performance decrements for pitch-proximal tone sequences as compared to pitch-distal sequences. Interestingly, this effect was observed only in non-musicians, whereas a group of musically trained participants showed only a phonological similarity effect but no pitch proximity effect (this finding has been explained with the use of more elaborated encoding techniques in musicians, including verbal labels for tones or contour patterns; Williamson et al., 2010a, pp. 171–172). In addition, the study also revealed that both verbal and tonal recall declined with increasing sequence length, suggesting similar capacity limitations in verbal and tonal short-term memory.

While the results of that study indicate similar storage mechanisms for verbal and tonal short-term memory (i.e., capacity limited acoustical representations), it is still unclear whether processing of speech and tones occurs in the same auditory storage loop or in two distinct phonological and tonal storage systems. There is some indication that articulatory suppression (whispering) interferes with the recall of both verbal and tonal sequences, suggesting a single storage articulatory rehearsal system for both types of auditory information (Williamson, 2008). On the other hand, it has been found that recognition memory for tonal and verbal stimuli may be sensitive to different types of auditory distractors (Williamson et al., 2010b). In that study, a visual-auditory recognition task was used in which verbal or tonal sequences were first presented visually (i.e., written letters or tones in musical notation format) and a comparison sequence was presented aurally after a retention interval of 10 s. All participants were musically trained and their task was to indicate whether the visual and auditory sequences were the same or different. The authors found that accuracy in the verbal task was disrupted must by the presence of irrelevant speech (utterances of “one,” “two,” “three” produced by three different speakers), whereas accuracy in the tonal task was disrupted most by the presence of irrelevant tones (notes C3-B5 played by three different instruments). While these domain-specific forms of auditory distraction support the existence of two distinct storage systems for verbal and tonal stimuli (the authors noted that results could also be accounted for in terms of the similarity of physical characteristics during encoding), it is still unclear whether such a dissociation can also be found in musically untrained participants and in serial recall tasks (as compared the recognition task used by Williamson et al., 2010b). Therefore, in the present study, the specific irrelevant sound effects produced by speech and music were investigated (a) in both musicians and non-musicians and (b) in both a verbal and tonal serial recall task (adapted from Williamson et al., 2010a) that was shown to not require prior musical training or the ability to read music notation as in the case of the tonal visual-auditory recognition task.

The present study is an attempt to demonstrate specific effects of verbal and musical auditory distractors on verbal and tonal serial recall in non-musicians and musicians. We used a paradigm that allows to test serial short-term memory for tonal sequences in both musically trained and untrained participants (Williamson et al., 2010a), but instead of manipulating pitch proximity (and phonological similarity), different types of task-irrelevant sound were presented while participants were encoding and maintaining sequences of pitch-proximal tones or phonologically similar letters (note that only proximal tones were used to maximize possible differences in tonal memory between musicians and non-musicians). In contrast to the simple and artificial sequences of irrelevant sound used in the previous study (random words or tones; Williamson et al., 2010b), more natural excerpts of classical instrumental music (taken from Salamé and Baddeley, 1989) and free-running speech in languages not spoken by the participants (used in previous studies; Zimmer et al., 2008; Kattner and Ellermeier, 2014) were presented as irrelevant sound in the present study. If verbal and tonal short-term memory were based on separate storage mechanisms, then irrelevant speech should interfere most with verbal serial recall, whereas irrelevant music should interfere most with tonal serial recall, regardless of musical experience.

In addition, we aim to replicate the previously reported sequence length effect for both verbal and tonal recall using the longer sequence lengths between five and seven items (Williamson et al., 2010a, b). The manipulation of the sequence length also allows to test whether the effects of irrelevant speech and music depend on the capacity limitations of verbal or tonal short-term memory. If participants were changing their encoding strategies with increasing sequence length and task difficulty (e.g., switching from phonological rehearsal to semantic encoding/chunking; Salamé and Baddeley, 1986; Hanley and Bakopoulou, 2003), then it could be expected that the degree of auditory distraction declines with increasing sequence lengths.

Finally, the comparison of verbal short-term memory between musicians and non-musicians allows to contribute to the debate on the relationship between musical training and cognitive skills. There is some evidence suggesting that trained musicians’ verbal memory performance is superior to that of non-musicians (e.g., Brandler and Rammsayer, 2003; Besson et al., 2007; Franklin et al., 2008; Jakobson et al., 2008), but most of these findings are restricted to small sample sizes, specific populations (e.g., children), or measures of long-term memory, and other researchers did not find group differences between musicians and non-musicians for verbal serial recall tasks (Williamson et al., 2010a). Hence, there does currently not seem to be sufficient evidence for verbal serial recall performance to depend on musical experience. On the other hand, in line with previous findings, we do expect musically trained participants to have higher tonal short-term memory spans than non-musicians.

## Materials And Methods

### Participants

A total of 50 participants (37 women) were recruited as participants at the campus of University of Hamburg. Twenty-seven of the participants (18 women) were self-reported non-musicians, who were unable to read music notation, and never had formal training on a musical instrument or singing for longer than 1 year (see Franklin et al., 2008). The non-musicians’ ages ranged between 18 and 55 years (*M* = 26.1; *SD* = 8.8). The remaining 23 participants (19 female) were musicians who were able to read music notation, had musical training (instrument or singing) for more than 7 years starting before the age of 10 years, and currently played or practiced an instrument (or were singing in a band or choir). The musicians’ ages ranged between 18 and 39 years (*M* = 23.3; *SD* = 4.7). The data of one additional musically trained participant who reported to have absolute pitch were not included in the analyses. All equalized participants gave written informed consent and were compensated with course credit.

### Apparatus and Stimuli

The experiment was conducted in a standard participant testing room at University of Hamburg. Sounds were generated at a sampling rate of 44.1 kHz (16 bits) using an Aureon 5.1 PCI sound card (Terratec, Alsdorf, Germany) and played diotically via a Philips SHM7410U (320 ohm) headset. The experimental routines were programmed in MATLAB (Mathworks, Natick, MA, United States) utilizing the Psychophysics toolbox extensions (Brainard, 1997; Pelli, 1997; Kleiner et al., 2007). Visual stimuli were presented on a 24-inch LCD monitor (Dell).

Three different sine tones with frequencies of 261.6 Hz (C4, “low”), 293.7 Hz (D4, “medium”), and 329.6 Hz (E4, “high”) were generated in MATLAB (corresponding to the pitch-proximal tones used by Williamson et al., 2010a). Each tone had a duration of 800 ms including cosine-shaped 20 ms rise- and fall times. For the tonal serial recall task, the tones were presented in sequences with adjacent tones being separated by 200 ms gaps of silence. Recordings of a male speaker uttering the letters B, G, and T were used as to-be-remembered items in the verbal serial recall task. Each recording had a duration of 1000 ms including short intervals of silence before and after the utterance. The intensity of each to-be-remembered sound (tones and letters) was RMS equalized, resulting in a sound pressure level (SPL) of approximately 72 dB.

Additional recordings of two different 14-s excerpts of classical instrumental music (Maurice Ravel’s “Bolero” and Kenneth J. Alford’s “Colonel Bogey March” from “Bridge over the River Kwai”; see Salamé and Baddeley, 1989), two different 14-s excepts of free-running speech in a language unknown to the participants (a Finnish weather forecast and a Korean poem both recited by male speakers and taken from Kattner and Ellermeier, 2014; Zimmer et al., 2008), and MATLAB-generated 14-s streams of white noise (which was shown to produce no disruption of serial recall compared to silence, but it can be matched with speech in terms of SPL; e.g., Salamé and Baddeley, 1989; Jones et al., 1990; Ellermeier and Zimmer, 1997; Ellermeier and Hellbrück, 1998) were used as irrelevant sound during encoding and retention of the tonal or verbal sequences. The irrelevant sounds were RMS equalized and played at approximately 66 dB SPL.

### Experimental Design

A 2 (group: musicians, non-musicians) × 2 (task: tonal or verbal serial recall) × 3 (sequence length: 5, 6, or 7) × 3 (irrelevant sound: noise, speech or music) mixed-factors design was implemented with task, sequence length, and irrelevant sound being manipulated within subjects.

### Procedure

The experiment started with a pre-exposure phase in which five-tone sequences consisting of random permutations of the three tones were played while the pitch of each tone (low, medium, high) was highlighted as filled circles from left to right in a 3 × 5 response grid shown on the screen. On the left end of the response grid, the three rows were labeled in German as “hoch” [high], “mittel” [medium], and “tief” [low]. Participants listened to five successive random sequences (and watched the response grid being filled) in order to familiarize with the low, medium, and high tones.

The main task of the experiment consisted of 54 tonal and 54 verbal serial recall trials. The two recall tasks were matched procedurally and adapted from a previous study demonstrating that tonal sequences can be recalled with this task above chance irrespective of musical training (Williamson et al., 2010a; more typical serial recall tasks with five to seven unique tones might be too difficult for non-musicians). In each task, sequences of either 5, 6, or 7 tones or spoken letters were drawn from a set of three unique items and presented sequentially while either white noise, speech (Korean or Finnish on half of the trials each), or music (Ravel or Alford on half of the trials each) was played as irrelevant sound. Each within-subjects condition (task × sequence length × irrelevant sound) was repeated six times throughout the experiment. The 108 trials were presented fully intermixed and in random order. Before starting with the actual task, participants were completed four additional practice trials with sequence length 5 and 7 both for the tonal and the verbal task. Each trial started with a 1-s preparation period in which the task was indicated with a text message (“Attend to the tones!” or “Attend to the letters!”) while a bluish square decreasing in size was presented in the center of the screen. A random sequence of tones or letters was then presented via headphones at a rate of one item per second while task-irrelevant sound was presented. The items were drawn randomly with replacement from the set of three and without the same item being repeated immediately. The sequence length varied between 5, 6, and 7, and the irrelevant sound continued after the last item until 14 s had passed. Subsequently, the visual response grid (3 × 5, 3 × 6, or 3 × 7, respectively) was shown on the screen and participants were asked to enter the sequence of tones or letters by clicking in the respective cells of the response grid. Depending on the task, the rows of the response grid were labeled either with “high,” “medium,” “low” or with “B,” “G,” “T” (from top to bottom). Participants could only click the cells from left to right without the possibility to correct their responses. After clicking the last item, feedback was presented on the screen for 1 s telling the number of correctly recalled items (e.g., “5 out of 7 correct!,” if more than half of the items were correct the feedback was presented in green font, otherwise in red font). The next trial started after a 250-ms blank-screen interval.

## Results

**FIGURE 1:**
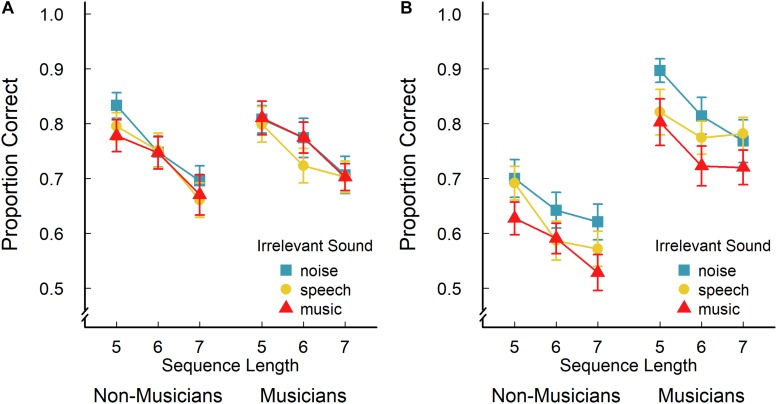
Non-musicians’ and musicians’ mean proportion of correctly recalled items in the verbal **(A)** and tonal **(B)** serial recall task as a function of the sequence length and irrelevant sound played during the encoding and retention interval. Error bars indicate ± 1 standard error of the mean.

The non-musicians’ and musicians’ average serial recall accuracy for verbal and tonal materials (i.e., the proportion of items recalled in the correct serial position) is illustrated in Figures 1A,B, respectively. A 2 (group) × 2 (task) × 3 (irrelevant sound) × 3 (sequence length) mixed-factors ANOVA with task, irrelevant sound, and sequence length as within-subjects factors revealed a significant main effect of task, *F*(1,48) = 8.14; *p* = 0.006; η_G_^2^ = 0.03: On average, performance (proportion correct) was better in the verbal serial recall task (*M* = 0.748; *SD* = 0.106) than in the tonal serial recall task (*M* = 0.696; *SD* = 0.154). The analysis also revealed a significant main effect of group, *F*(1,48) = 8.15; *p* = 0.006; η_G_^2^ = 0.06, with musicians showing the better overall performance (*M* = 0.772; *SD* = 0.101) than non-musicians (*M* = 0.680; *SD* = 0.099). However, there was an additional significant group × task interaction, *F*(1,48) = 18.78; *p* < 0.001; η_G_^2^ = 0.06, indicating that there was a clear group difference in the tonal recall task (*M*_musicians_ = 0.789; *SD*_musicians_ = 0.131 vs. *M*_*non–m*__*usicians*_ = 0.618; *SD*_non–__musicians_ = 0.128), *t*(48) = 4.67; *p* < 0.001., whereas musical experience did not seem to be related to performance in the verbal recall task (*M*_musicians_ = 0.742; *SD*_musicians_ = 0.108 vs. *M*_non–__musicians_ = 0.756; *SD*_non–__musicians_ = 0.106), *t*(48) = 0.45; *p* = 0.66.

In addition, general auditory distraction was confirmed by a significant main effect of irrelevant sound, *F*(2,96) = 11.14; *p* < 0.001; η_G_^2^ = 0.01, with performance decrements under conditions of irrelevant speech (*M* = 0.718; *SD* = 0.112) and music (*M* = 0.702; *SD* = 0.124), as compared to trials with white noise being played (*M* = 0.747; *SD* = 0.112).

**FIGURE 2:**
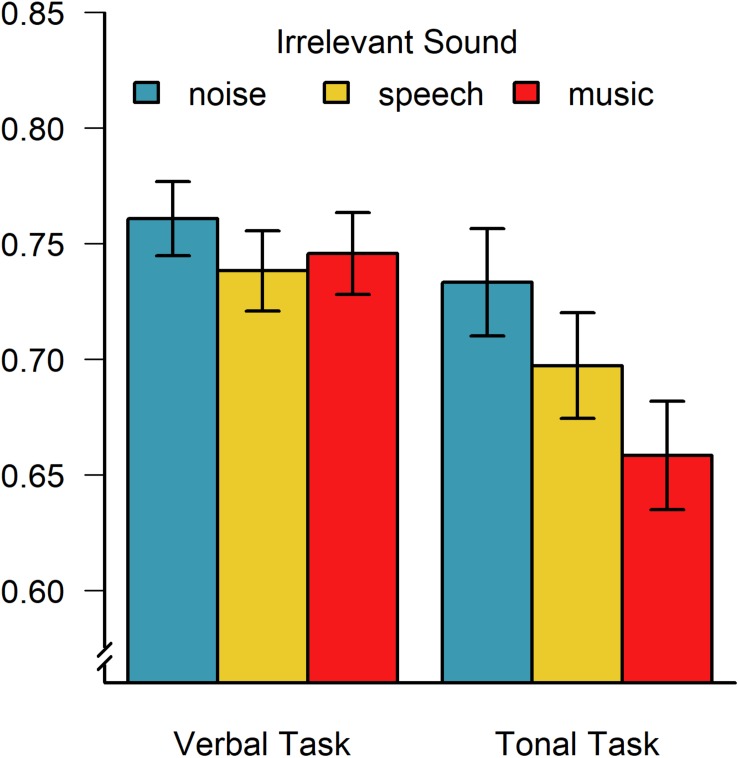
Average proportion of correctly recalled verbal and tonal items as a function of the type of irrelevant sound, demonstrating task-specific effects of irrelevant speech and music (collapsed across musicians and non-musicians). Error bars indicate ± 1 standard error of the mean.

Most importantly, however, the irrelevant sound effect was qualified by a significant sound × task interaction, *F*(2,96) = 5.08; *p* = 0.008; η_G_^2^ = 0.01, indicating that—across all participants—speech was most distracting in the verbal recall task (*M*_speech_ = 0.738; *SD*_speech_ = 0.122 for speech vs. *M*_noise_ = 0.761; *SD*_noise_ = 0.124 and *M*_music_ = 0.746; *SD*_music_ = 0.125), whereas music was most distracting in the tonal recall task (*M*_music_ = 0.659; *SD*_music_ = 0.166 vs. *M*_noise_ = 0.733; *SD*_noise_ = 0.165 and *M*_speech_ = 0.697; *SD*_speech_ = 0.161). This interaction indicates task-specific disruptions by phonological and tonal irrelevant sound, and it was independent of the degree of musical experience, as indicated by the absence of a three-way interaction with group, *F*(2,96) = 0.48; *p* = 0.62; η_G_^2^ < 0.01, and it is visualized in Figure 2 collapsed across musically trained and untrained participants. Additional one-sided paired *t*-tests confirmed that there was a significant difference in verbal recall performance between noise and speech, *t*(49) = 1.69; *p* < 0.05; *d*_*z*_ = 0.19, but not between noise and music, *t*(49) = 1.08; *p* = 0.14; *d*_*z*_ = 0.13, and not between music and speech, *t*(49) = 0.52; *p* = 0.70; *d*_*z*_ = 0.06. In contrast, for tonal recall performance, there was a significant difference between irrelevant noise and music, *t*(49) = 5.22; *p* < 0.001; *d*_*z*_ = 0.45, as well as between noise and speech, *t*(49) = 2.78; *p* = 0.004; *d*_*z*_ = 0.22, and the degree of distraction produced by irrelevant music was significantly larger than that produced by speech, *t*(49) = 2.96; *p* = 0.002; *d*_*z*_ = 0.24.

The ANOVA also revealed a main effect of sequence length, *F*(2,96) = 61.90; *p* < 0.001; η_G_^2^ = 0.07, with the proportion of correctly recalled items declining with increasing sequence length (*M*_5_ = 0.777; *SD*_5_ = 0.109; *M*_6_ = 0.717; *SD*_6_ = 0.113; *M*_7_ = 0.673; *SD*_7_ = 0.124), but there was no significant interaction between sequence length and irrelevant sound, *F*(4,192) = 0.49; *p* = 0.74; η_G_^2^ < 0.01, suggesting that the irrelevant sound effect did not change with increasing task difficulty. The analysis further revealed an only marginally significant interaction between sequence length and task, *F*(2,96) = 3.08; *p* = 0.05; η_G_^2^ < 0.01, indicating that effect of the sequence length may have been slightly more pronounced for verbal recall (*M*_5_ = 0.804; *SD*_5_ = 0.111; *M*_6_ = 0.753; *SD*_6_ = 0.120; *M*_7_ = 0.689; *SD*_7_ = 0.121) than for tonal recall (*M*_5_ = 0.750; *SD*_5_ = 0.164; *M*_6_ = 0.682; *SD*_6_ = 0.159; *M*_7_ = 0.658; *SD*_7_ = 0.165). The analysis did not reveal any other significant interactions, all *F* < 1.36; *p* > 0.24.

To further demonstrate the robustness of the abovementioned effects (which are based on only six data points per experimental condition and participant), hierarchical linear mixed-effects models were fitted to the data to account for random effects between and within participants (using the {lme4} package in R; Bates et al., 2015). Starting with a pure random-effects null model (with separate random intercepts fitted for each participant and trial, *AIC* = 1706), it was found that the addition of independent fixed effects of the four independent variables all successively improved the fit to the data, confirming significant main effects of sequence length, χ^2^(2) = 129.44; *p* < 0.001; *AIC* = 1581, group, χ^2^(1) = 10.05; *p* = 0.002; *AIC* = 1572, irrelevant sound, χ^2^(2) = 27.17; *p* < 0.001; *AIC* = 1549, and task, χ^2^(1) = 47.42; *p* < 0.001; *AIC* = 1504. Moreover, the addition of a group × task interaction improved the fit of the model considerably, χ^2^(1) = 106.43; *p* < 0.001; *AIC* = 1399, confirming the observation that musical experience influenced performance on the tonal task, but not on the serial task. Even more importantly, the addition of an interaction term for sound × task further improved the model fit; χ^2^(2) = 11.14; *p* = 0.004; *AIC* = 1392, supporting the assumption of differential effects of irrelevant speech and music on serial recall of verbal and tonal items (see Figure 2). Consistent with the above results, the addition of a three-way interaction term between sound, task, and group did not lead to further improvement of the model fit, χ^2^(4) = 1.63; *p* = 0.80; *AIC* = 1399, indicating that the task-specific effects of irrelevant speech and music did not depend on musical experience. Finally, the addition of a fixed effect for the sequence length × task interaction did also not significantly improve the model fit, χ^2^(2) = 4.16; *p* = 0.12; *AIC* = 1392, thus confirming the results of the ANOVA.

In addition to these frequentist statistics, Bayes factors were calculated (using the {BayesFactor} package for R; Rouder et al., 2009; Morey and Rouder, 2011) to estimate the likelihood of irrelevant music and speech to have an influence on serial recall of tonal and verbal items, respectively (relative to the null hypothesis of no difference between music/speech and noise). The Bayes factors (using a paired-observations design and a Cauchy prior distribution scaled with γ = 0.707) indicate that it is extremely likely that irrelevant music affected serial recall of tonal sequences, *BF*_10_ = 5345 (collapsed across groups and sequence lengths), whereas there was only moderate support for an irrelevant speech effect in the tonal recall task, *BF*_10_ = 3.49, suggesting specific disruptive effects of irrelevant music. In contrast, for the verbal recall task, there was no clear Bayesian support in favor of the relatively small effects of irrelevant speech (*BF*_10_ = 0.54), whereas irrelevant music was found to be four times more likely to have no effect than to disrupt verbal recall (*BF*_10_ = 0.25) in the present study (compare Figure 2).

## Discussion

The present study tested whether task-irrelevant music and speech interferes with short-term memory for tonal sequences. Specifically, we investigated whether irrelevant background music disrupts the serial recall of tonal sequences (i.e., short-term memory for melodies) more than other types of acoustical backgrounds such as speech and noise do. To that effect, we used a tonal recall task with random melodies consisting of five to seven tones (drawn with replacement from a set of three unique tones) which was previously found to allow both musicians and non-musicians to perform serial recall of tone sequences (Williamson et al., 2010a). Using an analogous verbal recall task, irrelevant speech (and to some extent also irrelevant music) was expected to disrupt the serial recall of verbal items (Colle and Welsh, 1976; Salamé and Baddeley, 1982).

We found that irrelevant music interfered with the serial recall of tone sequences in both musically trained and untrained participants, as compared to both background speech and noise. In contrast, serial recall of verbal items was disrupted only by the presence or irrelevant speech, but not by the presence of music or noise (in contrast to previous studies using slightly different recall tasks; e.g., Salamé and Baddeley, 1989; Nittono, 1997; Schlittmeier and Hellbrück, 2009; Avila et al., 2012). These task-specific disruptions produced by irrelevant music suggest that the retention of non-phonological tonal sequences may rely on the rehearsal of pitch contours (e.g., Berz, 1995), with any pitch changes contained in task-irrelevant music (or speech) gaining obligatory access to the “tonal rehearsal loop”—whereas phonological rehearsal is most likely used for the retention of verbal sequences in the “phonological loop” (i.e., using phonological rehearsal; Baddeley, 2003). Although musicians were generally better in the tonal recall task (which was found previously and is most likely due to experience with similar tonal tasks Williamson et al., 2010a), the specific interference between irrelevant music and tonal recall was observed in both groups (and of similar magnitude), suggesting that encoding and retention of tone sequences was based on the same mechanisms of auditory short-term memory. Hence, the present results indicate that both musicians and non-musicians were capable of rehearsing tonal sequences, and that the pitch contours in irrelevant music induces interference with tonal short-term memory irrespective of the degree of musical training.

In contrast, serial recall of verbal items in an analog letter recall task was found to be disrupted only by task-irrelevant speech (though with smaller effect size compared to the interference in serial recall of nine unique verbal items; Ellermeier and Zimmer, 1997), but not by task-irrelevant instrumental music. This irrelevant speech effect on verbal short-term memory was also observed regardless of musical experience and it suggests that the retention of verbal information relies on a separate phonological rehearsal process. Together, these task-specific effects of irrelevant music and speech on serial recall of tonal and verbal sequences, respectively, support the assumption of two separate auditory short-term memory systems for phonological and musical information (see Deutsch, 1970; Pechmann and Mohr, 1992; Berz, 1995). Specifically, serial recall of tonal sequences may be based on a pitch-based rehearsal system which is disrupted by the presence of irrelevant pitch information as contained in both music, and to some extent also in speech. In contrast, the phoneme-based short-term memory system (the phonological loop; Baddeley and Hitch, 1974; Baddeley, 2003) seems to be disrupted primarily by phonological distractors such as irrelevant speech and other types of changing-state sounds containing cues to the spectral or temporal modulations of speech (e.g., Ellermeier et al., 2015; Ueda et al., 2019).

However, we note that the phonological character of verbal short-term memory is being discussed controversially. For instance, articulatory suppression was found to eliminate the phonological similarity effect and the irrelevant speech effect not only with visual items, but also with auditory presentation of the to-be-remembered items, suggesting that the effect is driven by perceptual-motor processes during articulatory rehearsal rather than by processes of phonological storage (Jones et al., 2004; Exp. 3). It is thus possible that the to-be-remembered auditory items are not necessarily maintained though phonological rehearsal (representations in a phonological store), but rather through mapping of perceptual information (e.g., resulting from auditory stream segregation) on motor plans (e.g., articulatory movements) forming an episodic record of the stimuli (without assuming modality-specific storage systems; see Hughes et al., 2016).

In general, the pattern of auditory distraction observed in the verbal recall task is less consistent with the assumption of two separate mechanisms for verbal and tonal recall than the pattern observed in tonal recall task. Specifically, a dissociation between a verbal and tonal loop (e.g., Berz, 1995) would predict (1) music to be more disruptive than speech on tonal recall and (2) speech to be more disruptive than music on verbal recall. While the first prediction referring to tonal recall was clearly confirmed by both frequentist and Bayesian statistics, the second prediction was not supported consistently by the statistics (i.e., verbal recall under music did neither differ significantly from speech, whereas Bayes factor indicates that the disruptive effect of music is more likely to be equivalent to noise than the disruptive effect of speech). Hence, while irrelevant speech disrupted verbal recall, it is not entirely clear from the present study whether music reliably produces less disruption than speech on verbal recall. However, it has been reported previously that instrumental music is less disruptive than vocal music and speech for serial recall (e.g., Salamé and Baddeley, 1989), suggesting that phonological information produces additional interference in the phonological loop beyond the degree of changing-state information contained in instrumental music (see also the difference between staccato and legato music; Schlittmeier et al., 2008; Schlittmeier and Hellbrück, 2009). It is thus possible that the lack of evidence for a dissociation between the disruptive effects of music and speech on verbal recall may be due to the specific properties of the verbal recall task used in the present study which may have affected serial-order processing.

Interestingly, the magnitude of the irrelevant speech effect in the present verbal recall task was much smaller than in previous studies using a more typical serial recall task in which participants were memorizing random series of six (Kattner and Ellermeier, 2014), seven (Jones and Macken, 1993; Marsh et al., 2019), eight (Ellermeier et al., 2015; Kattner and Ellermeier, 2018; Bell et al., 2019), or nine unique verbal items (Jones and Macken, 1993; Ellermeier and Zimmer, 1997; Campbell et al., 2002). This discrepancy suggests that participants may not have used serial phonological rehearsal as the predominant strategy to perform the present verbal recall task in which series consisting of random permutations of only three unique verbal items (i.e., letters drawn randomly with replacement) were to be memorized. It is possible that participants instead memorized the contour pattern of the three letters (e.g., by visualizing the contour on a mental response grid, as it was used to enter the response in both tasks of the present study), which may be a more efficient strategy compared to serial rehearsal of the phonological sound of the entire sequence. In this case, it could be argued, in accordance with the modal working memory model (Baddeley and Hitch, 1974; Baddeley, 2003), that the letters were memorized using the visuospatial sketchpad, and task-irrelevant speech would thus not be expected to interfere with short-term memory (for a model and some evidence indicating that speech might interfere with serial recall even in this case, see Jones and Macken, 1993; Jones et al., 1995, 1996). Alternatively, it could be argued that the different types of utterances (i.e., the letters B, G, and T) form three distinct auditory streams in the participants’ perception, thus disturbing the perception of temporal order (similar alternating voices; see work on the talker variability effect; Hughes et al., 2011). In this case, auditory streaming could have impaired serial-order processing, and participants may have used recall strategies other than serial rehearsal (e.g., encoding the temporal pattern of each stream), thus potentially explaining the relatively small disruptive effects produced by irrelevant speech.

It could be speculated also whether the to-be-remembered tones were perceptually more difficult to separate from the streams of irrelevant music than to irrelevant speech (similar to a sandwich effect; Nicholls and Jones, 2002). Likewise, to-be-remembered spoken letters might be more difficult to separate from irrelevant speech than from irrelevant music. However, we do not consider this to be a likely explanation for the specific pattern of effects observed in the present study. In particular, the sinusoidal pure tones used for the tonal recall task (i.e., 261.6, 293.7, and 329.6 Hz) are expected to “pop out” from the background music of broader frequency spectrum (i.e., the excepts of classical music were compositions with multiple brass, woodwind, and string instruments conveying various wide frequency spectra that did not share many acoustical properties with pure tones), whereas the to-be-remembered verbal items may certainly share some speech-specific properties (e.g., syllable rate or individual phonemes) with free-running background speech (though it was in a foreign language). Hence, if the observed effects were due to a lack of perceptual segregation of relevant and irrelevant auditory streams, then the irrelevant speech effect on verbal recall should have been more pronounced than the effect of classical music on the recall of pure-tone sequences (which is the opposite to what was observed).

While musicians were able to recall more items than non-musicians in the tonal serial recall task, the present study did not reveal any group differences for the verbal serial recall task. Hence, the present results suggest that musical training is not related to verbal short-term memory capacity. This finding is consistent with a previous study using the same tonal recall paradigm in musicians and non-musicians (Williamson et al., 2010a), but it is not in line with studies that reported superior verbal memory and benefits for other cognitive abilities in musically trained individuals (Brandler and Rammsayer, 2003; Besson et al., 2007; Franklin et al., 2008; Jakobson et al., 2008). However, due to several procedural discrepancies between those studies and the present study, it is possible that the effects of musical training on verbal memory are restricted to early developmental stages (Besson et al., 2007) or to different types of memory measures (e.g., long-term memory; Franklin et al., 2008; Jakobson et al., 2008).

Taken together, the present study demonstrated that task-irrelevant music interferes specifically with the short-term recall of random melodies (sequences of pure tones), but not with the serial recall of verbal sequences (i.e., phonologically similar spoken consonants). This task-specific irrelevant music effect was found in both musically trained and musically untrained participants, suggesting that tonal short-term memory relies on the same pitch-based rehearsal mechanism (e.g., Berz, 1995) regardless of musical experience. In contrast, task-irrelevant free-running speech was found to interfere less with serial recall of tonal sequences, and it reliably disrupted serial recall of verbal items (as compared to noise), though to a lesser extent compared to previous studies in which participants are typically required to maintain a larger number of unique verbal items (e.g., Ellermeier and Zimmer, 1997; Campbell et al., 2002; Bell et al., 2019). The results indicate a functional dissociation between short-term memory for phonological and non-phonological auditory items which needs to be further investigated in future studies.

## Data Availability Statement

The datasets generated for this study are available on request to the corresponding author.

## Ethics Statement

Ethical review and approval was not required for the study on human participants in accordance with the local legislation and institutional requirements. The patients/participants provided their written informed consent to participate in this study.

## Author Contributions

FK and HM developed the study concept and designed the experiments. FK programmed the tasks and drafted the manuscript. HM recruited the participants and conducted the experiments. Both authors contributed to the data analysis and made several revisions.

## Conflict of Interest

The authors declare that the research was conducted in the absence of any commercial or financial relationships that could be construed as a potential conflict of interest.
